# Disentangling the relative importance of spatio-temporal parameters and host specificity in shaping arbuscular mycorrhizal fungus communities in a temperate forest

**DOI:** 10.1007/s00572-021-01041-6

**Published:** 2021-07-19

**Authors:** Leonie Grünfeld, Magkdi Mola, Monika Wulf, Stefan Hempel, Stavros D. Veresoglou

**Affiliations:** 1 Institut Für Biologie, Freie Universität Berlin, Altensteinstr. 6, 14195 Berlin, Germany; 2 Berlin-Brandenburg Institute of Advanced Biodiversity Research, 14195 Berlin, Germany; 3 Research Area 2, Leibniz Centre for Agricultural Landscape Research (ZALF), Eberswalder Straße 84, 15374 Müncheberg, Germany; 4 School of Ecology, State Key Laboratory of Biocontrol, Sun Yat-Sen University, Guangzhou, 510006 China

**Keywords:** Arbuscular mycorrhizal fungi, Host specificity, Herbaceous understory, Spatio-temporal dynamics, Stochastic vs. deterministic drivers, Temperate forests

## Abstract

Many woody and herbaceous plants in temperate forests cannot establish and survive in the absence of mycorrhizal associations. Most temperate forests are dominated by ectomycorrhizal woody plant species, which implies that the carrying capacity of the habitat for arbuscular mycorrhizal fungi (AMF) is relatively low and AMF could in some cases experience a limitation of propagules. Here we address how the AMF community composition varied in a small temperate forest site in Germany in relation to time, space, two plant host species, and also with regard to the degree to which plots were covered with AMF-associating woody species. The AMF communities in our study were non-random. We observed that space had a greater impact on fungal community composition than either time, mycorrhizal state of the close-by woody species, or the identity of the host plant. The identity of the host plant was the only parameter that modified AMF richness in the roots. The set of parameters which we addressed has rarely been studied together, and the resulting ranking could ease prioritizing some of them to be included in future surveys. AMF are crucial for the establishment of understory plants in temperate forests, making it desirable to further explore how they vary in time and space.

**Supplementary information:**

The online version contains supplementary material available at 10.1007/s00572-021-01041-6.

## Introduction

Arbuscular mycorrhizal fungi (AMF; Phylum Glomeromycota) are globally distributed symbiotic fungi, which at large spatial scales show non-random distribution patterns, explained mainly by abiotic predictors such as pH (Dumbrell et al. 2010a; Davison et al. 2021), soil properties (Klichowska et al. 2019), and climatic conditions (Dumbrell et al. 2011) but also host specificity (Vandenkoornhuyse et al. 2002). Most studies addressing AMF, however, can only explain a small fraction of AMF community variance, suggesting that AMF communities are subject to a high degree of stochasticity (i.e., the fraction of community variance not explained by deterministic processes; Supplementary Information, Appendix I; Dumbrell et al. 2010b; Lekberg et al. 2012; Goldmann et al. 2020). Assaying stochastic drivers in large-scale mycorrhizal studies is challenging because they can inflate the sequencing effort required. We here aimed at ranking the relative importance, in terms of shaping arbuscular mycorrhizal fungal communities, of a set of stochastic (space, time) and biotic (host plant species and woody coverage of AMF-associating species) drivers rarely assayed together, in an effort to smooth the way towards integrating stochastic predictors into mainstream studies of mycorrhizal fungus communities.

We present a spatio-temporal study at a forest site where we address the relative importance of (i) physical distance, (ii) sampling time (year and season), (iii) host specificity (here describing the impact of host plant identity on the AMF community composition in the roots of focal plant species), and (iv) relative coverage of AMF-associating woody species (AM *high* and *low* plots, which have been shown to differ in regards to their AMF dynamics: Veresoglou et al. 2017; Grünfeld et al. 2019) in shaping the AMF community composition of the understory. To the best of our understanding, no other mycorrhizal study to date has simultaneously studied this specific set of parameters, even though studying subsets of them have generated highly valued expectations: Davison et al. (2012), for example, studied the effects of seasonality and spatial structure in an Estonian temperate forest and observed considerable spatial heterogeneity in AMF species distributions, but minimal changes over the duration of a growth season. Dumbrell et al. (2011), by contrast, observed pronounced temporal changes in the composition of AMF grassland root communities over a single growth season. Su et al. (2011) addressed the relative strength of host specificity and seasonality to show that in the studied steppe of Inner Mongolia, seasonality masked any host preferences across five hosts. Therefore, our first expectation (*Hypothesis 1*) was that physical distance would be relatively more important than temporal variance in shaping AMF communities in a woody habitat (Davison et al. 2012). It is likely that woody plants in such studies had strong effects on the understory because they had acted as islands of AMF propagules (Grünfeld et al. 2019). If it is the presence of AMF-associating woody species which mainly shapes the regional pool of AMF species, then compared to grasslands, we might expect a lower relative importance of host specificity across the understory plants because tree root systems are comparably much larger than those of understorey plants. AMF propagule selectivity among hosts (i.e., which might lead to the evolution of host dependency on specific fungus species) in the understory hence is determined to a large degree by the identity of the neighbouring woody AMF-associates. We therefore additionally hypothesized (*Hypothesis 2*) that relative coverage of AMF-associating woody species would alter AMF community composition more than host specificity does. We addressed these two hypotheses in a forest site in the Elbe-Weser region in North-West Germany which we monitored over 2 years, totalling four harvests of root material.

## Materials and methods

### Study site

The study site is a floristically well described (Wulf 1992; Naaf and Wulf 2010) temperate European deciduous forest in northwest Germany (53.44°N, 9.49°E). The soil is a humid to waterlogged pseudogley. Biophysical characteristics were assumed to be relatively consistent over the homogeneous 625-m^2^ site, and therefore, we did not explicitly measure or include them in this study. Nevertheless, we report regarding previously assessed soil parameters at the same site by Wulf (1992) in the supplements (see Table S2). Based on previous observations in the broader area, understory plants associating with AMF occurred at higher frequency (Veresoglou et al. 2017) and were colonized more extensively by AMF (Grünfeld et al. 2019) when there was a high relative coverage of woody plants forming arbuscular mycorrhizae. It is likely that the occurrence and density of arbuscular mycorrhizal (AM) woody plants facilitate the dispersal of AMF propagules and thus their availability to less dominant (with respect to biomass) understorey AM plants. To assess AMF community variability related to AM woody coverage, we divided the forest site into 25 5- × 5-m rectangular plots and estimated in situ coverage by AMF-associating woody plants per plot. AM woody coverage ranged from 0 to 60%, and we subsequently classified the plots into AM *high* and AM *low* classes (≥ 15% and < 15% AMF-associating woody coverage, respectively; Fig. S1, Table S1; we rationalize the choice of the threshold in Fig. S2).

### Sampling design

Over 2 years, we collected root samples from the two most abundant perennial woody understorey plant species *Hedera helix* L. (Araliaceae, from now on *Hedera*) and *Euonymus europaeus* L. (Celastraceae, from now on *Euonymus*) in the forest site. In the beginning (May) and end (September) of the growing seasons of 2017 and 2018, respectively, we collected *Hedera* roots (78 samples) from pairs (i.e., two neighbouring plots of high and low relative coverage of AMF-associating woody species) of *high* and *low* AM plots. In September 2018, we additionally collected roots of *Euonymus* (19 samples; *Euonymus* could only be collected at the last harvest because there were only a few individuals of *Euonymus* in the forest site and their destructive harvest could modify meta-community dynamics of AMF species.). The two hosts were sampled independently of each other, meaning that *Euonymus* and *Hedera* separated by less than 50 cm potentially could have been sampled. Because both hosts were woody species, we expected them to phenologically vary less in time than herbaceous plants. Rarely, two individuals of a species were not available in a plot. Thus, there were a total of 97 root samples from the two host plants from the four sampling campaigns (see Table 1 and Fig. S1 for the specific sampling scheme). Roots were excavated to a maximum depth of about 10 cm from two plant individuals of each focal species per plot, which were processed independently. Assaying a depth of 0–10 cm maximized compatibility of our findings with the bulk of the literature and did not cause excessive disturbance to the forest site. The minimal distance between the two individuals of the same plant species was 50 cm to minimize the likelihood that the two root samples shared AMF individuals (Klironomos and Moutoglis 1999). The root samples were cleaned with water and transferred into falcon tubes with 95% ethanol.

**Table 1:** Sampling scheme showing the number of samples (*n*) and plots (*n*_plots_) per sampling campaign and plant species

	May 2017	Sep 2017	May 2018	Sep 2018
*Hedera helix*	*n* = 12 *n*_plots_ = 6	*n* = 20 *n*_plots_ = 10	*n* = 20 *n*_plots_ = 10	*n* = 26 *n*_plots_ = 14
*Eunonymus europaea*	Not sampled	Not sampled	Not sampled	*n* = 19 *n*_plots_ = 10

### Molecular analyses and bioinformatics

Roots were transported to the lab ln ethanol at 4 °C and stored at −20 °C. Root samples were freeze-dried and homogenized with a Retsch Mixer Mill MM 400 using metal balls of 1-mm diameter. DNA was extracted from 30 mg ground root material per sample with the DNeasy® PowerPlant® Pro Kit (Qiagen, Venlo, the Netherlands) and amplified with the AMF-specific-18S-rRNA gene targeting primer pair NS31-AML2 (Liu et al. 2011) extended with the adaptors p5 (NS31) and p7 (AML2; Kircher et al. 2012). The amplification conditions were as follows: each of the 25 µl PCR reactions contained 1 µl DNA template, 2.5 µl (0.3 µM of each primer) primer mix, 0.25 µl KAPA HiFi DNA polymerase (1 U/µl), 0.5 µl KAPA dNTP mix (10 µM), 5 µl 5X KAPA HiFi Fidelity Buffer, and 15.75 µl nuclease-free water. The PCR reactions were performed with a Biometra-Ton thermal cycler (Analytik Jena, Jena, Germany) under the following conditions: Initial denaturation at 95 °C for 2 min, 35 cycles with a denaturation phase of 98 °C for 45 s, an annealing phase of 65 °C for 45 s, and an extension phase of 72 °C for 45 s and final elongation at 72 °C for 10 min. Samples that did not perform well on this initial PCR (~ 40% of the samples) were amplified instead with a GC-rich buffer from the kit. Four out of 97 samples did not show bands during gel electrophoresis and were excluded from further analysis. The NS31-AML2 amplicons were purified with the NucleoSpin® gel and PCR clean-up kit (Macherey–Nagel, Düren, Germany). For indexing purposes, we used Miseq specific adaptors (NuGen) which we ligated to our products with an additional PCR. The PCR master mix for indexing consisted of 1 µl of the purified PCR template, 2.4 µl of the primer mix, 0.25 µl Phusion® high-fidelity DNA polymerase (BioLabs), 0.5 µl dNTPs (10 µM), 5 µl 5X Phusion® HF buffer, and 15.85 µl nuclease-free water per 25 µl reaction. After indexing PCR—thermocycling settings: 95 °C for 3 min, 15 cycles of 98 °C for 30 s, 55 °C for 30 s and 72 °C for 30 s, and 72 °C for 5 min—the DNA fragments were separated by gel electrophoresis to check the signal strengths. We used MiSeq Illumina chemistry (v3, 600 cycles) to sequence the amplicons. We processed the libraries with the uPARSE pipeline (Edgar 2013) with uSearch v 10.0.240. In brief, forward and backward reads were merged with the fastq_mergepairs command, primers were stripped and sequence pairs with a length shorter than 400 bp, or more than 1 expected error were removed. We used the cluster_otus command to construct the OTU table. Representative OTU sequences were blasted against MaarjAM (Öpik et al. 2010) and non-specific to AMF OTUs (i.e., < 97.5% similarity or < 99% coverage) were excluded from further analyses. Representative sequences for each OTU were submitted to GenBank (submission MW017500-MW017533). We then rarefied to 2350 reads per sample, which excluded 7 samples from further analysis (i.e., analysis was carried out to the remaining 90 samples; 73 described communities in roots of *Hedera* and 17 AMF communities in the rooty of *Euonymous*).

### Statistical analyses

#### Null model analysis: to what degree were AMF distributions random?

To address the degree to which AMF communities were random, we conducted a null model analysis with the R package EcoSimR (Gotelli et al. 2015). We compared C score occurrences in our dataset to distributions of 1000 random matrices that were generated with the *sim*4 algorithm. C score occurrences of checkerboards describe the cumulative number of occurrences across a pair of sites (= rows) and species (= columns) in the presence-absence community matrix where Species A has only been present at Site A and Species B has only been present at Site B. We z-score standardized effect sizes (SES) in relation to the set of simulated community matrices. We used the presence-absence data and kept the total number of row sums in the community table fixed, describing how often species occurred. The row sums were proportional to those observed in the column sums, reflecting differences across samples. The *sim*4 algorithm effectively controls for Type I and II statistical errors and has been proposed for scenarios in which some rare species occasionally have been scored as absent even though present (i.e., incomplete lists; Gotelli 2000). Negative standardized effect sizes below −1.96 reflect aggregation of species within samples (= fungal species co-occur more often than expected by chance), positive values above 1.96 reflect segregation of species within samples (= fungal species co-occur less often than expected by chance), whereas values between −1.96 and 1.96 reflect a random species distribution among plots. Additionally, values differing by more than 1.96 standardized units were significantly different (analogous to a confidence interval). Because inadvertent pooling of heterogeneous samples (due to combining in the same analysis root samples differing in time, space, and also plant host) might bias results towards appearing less random (Ulrich et al. 2012), we additionally assessed null model statistics for several subsets of the combined community matrix.

#### Hypothesis 1: Physical distance is more important than temporal variance in structuring AMF

Because our study design was complex and difficult to be fully captured with statistical techniques, we tried whenever possible (such as in Fig. 2) to present effect sizes which assumed no specific statistical model. To address this hypothesis, we (i) visualized the raw data via unconstrained ordination, (ii) calculated effect sizes in the form of Bray–Curtis community distances for the major drivers of AMF community composition, and (iii) presented as a key result a summary for some characteristic groups of samples of community composition information at the AMF family level. First, we carried out a principal components analysis (PCA) on Hellinger-transformed AMF OTU occurrence data (i.e., AMF community table with each OTU treated as an independent response variable) to visualize clustering patterns across the samples. Second, we presented how effect sizes differed among our variables of interest. We wanted to avoid statistical shortcomings of combining a redundancy analysis (i.e., a form of constrained ordination) with variance partitioning. Even though there are several techniques to address spatial autocorrelation in ordination analyses, to the best of our knowledge, the only multivariate technique that works for temporal constraints is that of Palmer et al. (2008) which was specifically proposed for split-plot designs. To minimize the assumptions of our analyses, we plotted the data with a PCA (i.e., meaning that we do not propose for this specific analysis any underlying model; Fig. 2a) and then calculated the distributions of pairwise Bray–Curtis distances. We visualized relative effect sizes by means of Bray–Curtis distances and only additionally fitted a predictive model in the form of a redundancy analysis (RDA) in which we addressed temporal constraints by restricting permutations (and thus calculation of resulting *P* values) to be only within plots. We further decomposed distance (i.e., spatial) information into three principal coordinate neighbouring matrices (PCNM; Borcard and Legendre 2002) which we then fitted into the RDA model. This approach may be an improvement compared to assuming full independence, but it still falls short of describing our spatio-temporal sampling design. For this reason, we cautiously interpreted the resulting variance partitioning exercise. To compare effect sizes, we randomly paired samples sharing specific attributes 9999 times and quantified Bray–Curtis distances. Third, we summarized how AMF community composition differed with each of the predictors by generating bar plots with relative abundance information on each AMF family. We finally created a heatmap (i.e., a two-dimension graphical representation of community data) presenting the frequencies with which individual AMF taxa were observed in habitats with specific attributes.

#### Hypothesis 2: Relative coverage of AMF-associating woody species would alter AMF community composition more than host specificity does

We first carried out a repeated-measures ANOVA to compare diversity metrics (i.e., richness, Shannon diversity, and Pielou evenness; in the Results section, we only report on richness but the results were comparable across all those diversity indices) between AM *high* and *low* plots. The response variable was the diversity metric; host species and *low* vs. *high* type of habitat were the predictors and time was the repeated measures parameter. In our repeated-measures ANOVA, we corrected for spatial dependencies in the form of specifying the unit of the ANOVA analysis at the “plot” level. To address whether the communities in *high* and *low* plots differed in relation to how aggregated/segregated they were, we further compared the respective SES which we obtained from our null model analysis. We created a Venn diagram depicting how host specificity and relative coverage of AMF-associating woody species influence AMF community composition to visualize compositional differences. To further address whether host plants or the two habitat types selected for specific OTUs, we finally carried out an indicator species analysis (we used the package indicspecies in R; De Cáceres and Legendre 2009)  in relation to the following classes: the two host plants, the two habitat types (i.e., *high* vs *low*) and their meaningful combinations.

## Results

### Overall statistics

Out of 853,811 quality-controlled reads, 696,451 described 33 AMF-specific OTUs (Table S3). Eighteen of them belonged to Glomeraceae, six to Claroideoglomeraceae, five to Archaeosporaceae, two to Diversisporaceae, and one each to Gigasporaceae and Acaulosporaceae. We rarefied sequencing depth to 2350 reads per sample which resulted in the exclusion of two samples. AMF richness ranged from 4 to 17 OTUs per sample (median: 10 OTUs with the quartiles being 8 and 12; Fig. S3). Richness only differed with host plant (*n* = 93, *t* =  − 4.44, *P* < 0.0001; when we narrowed observations to those from the fourth harvest, the respective statistics were *n* = 43, *t* =  − 3.14, *P* = 0.003; Fig. S3): *Euonymus* plants contained on average 8.2 AMF taxa, whereas *Hedera* plants contained 10.54.

The indicator species analysis classified 5 of the 33 species as indicators. OTU2 (Glomeraceae; *P* = 0.045) was an indicator of *Euonymus* communities and OTU70 (Glomeraceae, *P* < 0.001) an indicator of *Euonymus* community at *low* plots. OTU8 (Claroideoglomaceae; *P* = 0.001) and OTU13 (Acaulosporaceae, *P* < 0.01) were indicators of *Hedera* communities, whereas OTU19 (Diversisporaceae; *P* = 0.038) specifically associated with *Hedera* at *high* plots.

### Null model analysis: to what degree were AMF distributions random?

**Fig. 1:**
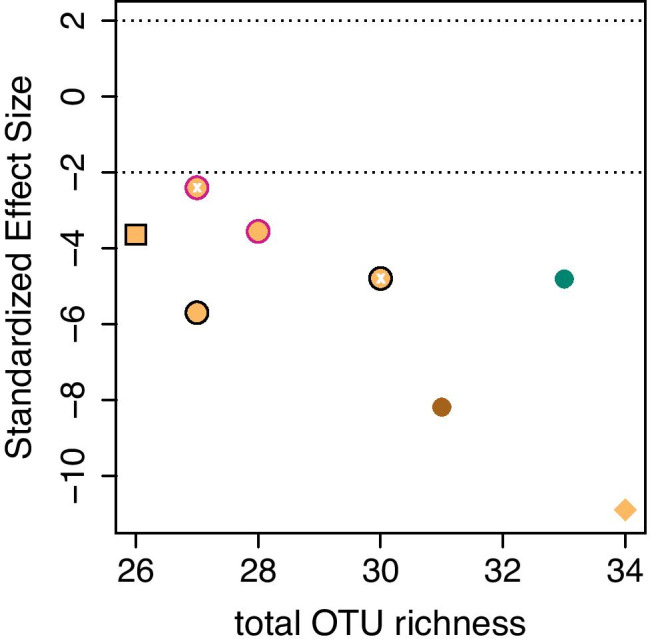
Standardized effect sizes of observed checkerboard scores which were compared against a null model generated with the sim4 algorithm (*y*-axis). We plotted these values against total OTU richness of the respective subsets of the dataset to capture a factor that may influence them. The two discontinuous lines highlight confidence intervals within which the community matrix can be considered random. The green point represents samples from AM *high* plots (> 15% woody AM-associating plant coverage), the brown from AM *low* plots (< 15% woody AM-associating plant coverage), and the orange points from combinations of the two. A pink border was used for spring and black for autumn; we used no border where we pooled samples from spring and autumn. We used white “x” symbols to highlight the location in the panel of samples taken over the first year. The square represents samples on *Euonymus* whereas circles those on *Hedera*. The diamond shows the complete data set. Differences in standardized effect sizes above 1.96 and below −1.96 are significant at a 0.05% confidence level

In all our tests, we observed significant species aggregation (Fig. 1). The standardized effect sizes (SES) ranged from −10.90 (combined community matrix) to −2.4 (*Hedera* roots in May 2017). AMF communities in *Hedera* roots from *low* plots (SES =  −8.19) were more aggregated than those from *high* plots (SES =  −4.81; any differences in the statistics exceeding 1.96 are significant). AMF communities in *Hedera* were more aggregated in autumn than in spring (the mean SES statistic for spring was −2.98, whereas for autumn, it was −5.25). The results in SES statistics could not be explained based on sampling intensity (i.e., number of individuals assayed; there was no correlation between the two values).

### Hypothesis 1: Physical distance is more important than temporal variance in structuring AMF

**Fig. 2:**
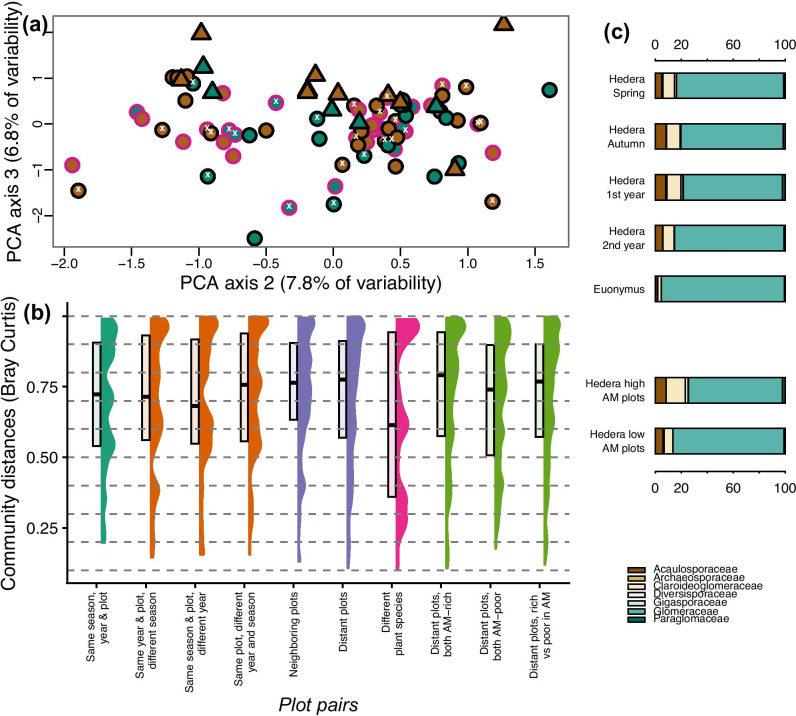
**a** Principal component analysis of Hellinger-transformed AMF community data (we plotted respective diagrams with axis one, explaining 13.5% of variability in Figs. S4 and S5). Green symbols represent samples from AM *high* (> 15% woody AM-associating plant coverage), whereas brown represent AM *low* plots (< 15% woody AM-associating plant coverage). A pink border was used for spring and a black for autumn. We used white “x” symbols to highlight the location in the panel of samples taken over the first year. Triangles describe samples on *Euonymus*, whereas circles those on *Hedera*. **b** Distributions of pairwise community distances (Bray–Curtis distances) for a range of pairwise combinations (dark green: within plots sampled at the same time; orange: same plot differing in sampling time; purple: same harvest but different plot; pink: same plot in the 4th harvest but different host plant; light green: same harvest but different plot grouped based on the relative coverage of AMF-associating woody plants). Larger values signify more dissimilar samples, meaning that the responsible factor induced a stronger AMF community shift than in the case of smaller values. As an example, the pairs belonging on the same plot which are presented in the four first histograms (in dark green and orange) consistently showed smaller values than those across different plots (two purples histograms) suggesting that space played a role in shaping AMF communities. Note that Bray–Curtis community distances between *Hedera* and *Euonymus* (in pink; same plot) were smaller than respective distances between individuals of *Hedera* (dark green). **c** Mean relative abundances of the seven AMF families (Acaulosporaceae, Archaeosporaceae, Claroideoglomeraceae, Diversisporaceae, Gigasporaceae, Glomeraceae, Paraglomaceae) grouped based on (top) the time of sampling, plant host, and (bottom) our classification into high and low plots

Our principal components analysis on Hellinger-transformed occurrence data showed that any differences in AMF community composition across the samples were so subtle as to be little apparent (Fig. 2a). We plotted axes two and three because after excluding an outlier sample, these two axes explained most rescaled variance. The take home message from the panel is that there were no apparent clustering patterns in our dataset against any parameter and any AMF community shifts in time or space thus were relatively small. The Bray–Curtis distributions overlapped considerably but spatial structure induced stronger effect sizes than temporal variability (Fig. 2b). In addition, community changes within a growth season were subtle (Fig. 2b). We also observed that the two host plant species (Fig. 2b) shared more similar communities than expected by chance and that it was low plots that had the most divergent AMF communities. (Fig. 2c). *Euonymus*-associated AMF communities were dominated by Glomeraceae (94.8% on average compared to a maximum of 86% in *Hedera*; Fig. 2c). High occurrence of Glomeraceae was also observed at low AM plots (averaging 85.5%). Relative abundance differences of families were considerably more pronounced across years than across seasons (Fig. 2c). In the redundancy analysis with the drivers as predictors, we found that year, host plant and spatial autocorrelation axes explained AMF community shifts, whereas season had no effect. AM-plant cover shared considerable variance with other predictors and significance depended on the ranking with which it was included among the predictors (Supplementary Information, Appendix III).

### Hypothesis 2: Relative coverage of AMF-associating woody species would alter AMF community composition more than host specificity does

**Fig. 3:**
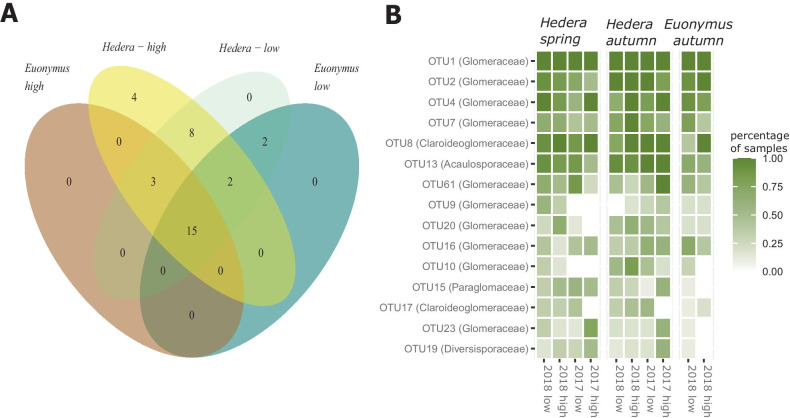
**A** Venn diagram depicting the distribution of OTUs across (i) AM *high* (> 15% woody AM-associating plant coverage) and AM *low* plots (< 15% woody AM-associating plant coverage) and (ii) the two plant hosts. Fifteen out of the thirty-two OTUs were observed in all four types of habitats. **B** Frequency of occurrence of the fifteen most abundant OTUs across ten groups of samples describing plant host, plot quality in relation to AMF abundance, and season of sampling

We observed no diversity differences in relation to *high* or *low* relative coverage of AMF-associating woody species (Fig. 1; F_1,81_ = 0.048, *P* = 0.83; the only significant effect was that of host plant; F_1,81_ = 11.8, *P* < 0.001). Roots from *low* plots contained consistently more aggregated AMF communities than the representatives from *high* plots (Fig. 1). There were minor compositional distances between *high* and *low* plots with 7 OTUs being specific to *high* plots and 2 to low plots (Fig. 3A). We observed, by contrast, twelve OTUs to be specific to *Hedera* samples (Fig. 3A), which might have been because of the most extensive sampling of *Hedera* individuals. Observation frequency, for most taxa, was higher at *high* plots than at *low* plots (Fig. 3B).

### Ranking of spatio-temporal parameters and host specificity

**Fig. 4:**
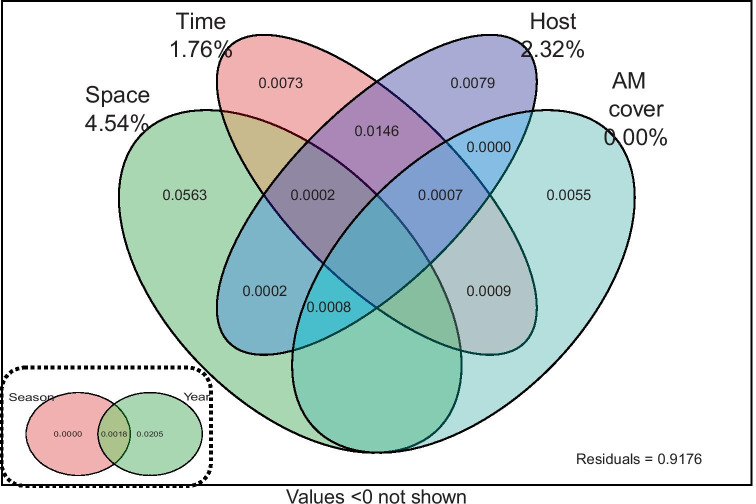
Partitioning of variance explained by spatial, temporal, host specific, and AM plant cover related parameters across AMF communities in our forest site. Spatial parameters comprised three PCNM axes, and temporal parameters comprised the effects of season (i.e. explaining zero variance; insert at the bottom left) and year. The estimates are biased and are presented only for comparative purposes: for example, the impact of host effects on AMF community structure should have been considerably higher than shown, but because we harvested *Euonymus* only once the parameter explained a relatively small part of the total variance. The variance partitioning additionally unrealistically assumes a completely balanced design with an equal representation of samples on all plots and invariable sampling effort across the four harvests. By including parameters that explained no variance such as season (insert at the bottom left), we further biased estimates. Finally, the analysis also does not capture that some plots have been assayed more than once and thus are not independent samples

Based on the variance partitioning exercise (Fig. 4), spatial parameters (4.54%) explained most variance followed by host specificity (2.32%). This was despite that the representation of hosts was unbalanced, meaning that the variance fraction allocated to host specificity actually should have been considerably larger. Temporal parameters explained 1.76% of the variance, but this fraction was exclusively due to different years and not due to different seasons (Fig. 4, insert). The relative coverage of AMF-associating woody species (i.e., AMF cover in Fig. 4) explained no variance. These observations match well the results from Fig. 2b.

## Discussion

A take home message of our study is that, in agreement with *Hypothesis 1*, physical distance in the studied temperate forest exerts a stronger influence on AMF communities than either sampling time or host specificity. We also show that temporal variability is slightly higher across years than across seasons. Hence, our data agree with Davison et al. (2012) that there is low seasonality in forests in relation to AMF communities. The order of establishment of plant hosts, known as priority effects, could thus play an important role in structuring AMF communities (Hausmann and Hawkes 2009). In natural systems, this most likely occurs at the beginning of the growing season. Even though this idea remains underexplored, it could potentially explain why the effect sizes for different years were larger than for different seasons.

Through our null model analysis, we deduced that the plant root AMF colonization patterns in our study had been non-random (even though AMF community differences with time, space, and hosts were weak; Fig. 2a) and showed extensive aggregation of species, meaning that the OTUs co-occurred more often than expected by chance. That our null model analysis supported that AMF root community composition was not random was not surprising (e.g., Hu et al. 2019). The outcome of co-occurrence analyses, however, depends strongly on how heterogenous the compared communities are (but also on sampling intensity): relatively homogenous communities such as those in our study are more likely to show aggregation, whereas heterogeneous pools of samples such as those analysed with a comparable approach in Hu et al. (2019) are more likely to show segregation. It was important in our study to first show that the community matrix at the employed spatial scale is non-random (and thus, our study had enough resolution to address community variance patterns in AMF communities), before addressing how spatio-temporal parameters and host specificity explained the community variance. Additionally, through our null model analyses, we could observe some overarching patterns such as that *low* plots hosted more aggregated AMF communities than *high* plots. Species aggregation patterns often suggest shared habitat requirements across species compared to mechanisms such as competition and dispersal limitation which induce segregation (e.g. Cordero and Jackson 2019). Thus, we might expect aggregating AMF taxa colonizing *Hedera* roots in *low* plots to have higher dispersal, but fewer competitive characteristics compared to communities on *high* plots.

In our RDAs, we observed pronounced plant host effects on AMF richness (Fig. 1), AMF community aggregation (Fig. 1), and community composition (Fig. 2c). The present study obviously did not fully address the role of host specificity: we only assayed two host plants, and because of the low abundance of *Euonymus*, we only assayed individuals at the last harvest. This mainly served the purpose of showing the degree to which our observations with *Hedera* corresponded to those with *Euonymus*. It nevertheless is likely that we could still get a reasonable (and hopefully representative) picture of how host specificity influences AMF communities. We present evidence, for example, that host specificity has a strong influence on AMF richness (i.e., plant host was the only parameter in our analyses that had an effect on AMF richness). We found of special interest, however, that pairwise differences between species (*Hedera*- vs. *Euonymus*-associating AMF communities; Fig. 2b) were smaller than respective pairwise differences of conspecific individuals (randomly paired in RDA models). There is evidence that phylogenetically divergent co-occurring plant species share more similar AMF communities than closely related species (Veresoglou and Rillig 2014) and our analysis hints towards that. Remarkably, most studies that have been carried out at a regional or global scale have found no evidence for host specificity (e.g., Davison et al. 2015). This could mean that abiotic conditions mask host specificity at scales larger than that of the present study. Alternatively, inconspicuous factors at a smaller scale (i.e., such as that in the present study) driven by the environment such as priority effects or the availability of AMF propagules could modify how plant species select for AMF communities.

Contrary to our expectations that *low* and *high* plots would host distinct AMF communities (*Hypothesis 2*), we only observed small associated differences in diversity, and the factor AM plant cover in the RDA was only conditionally significant (Supplementary Information, Appendix III). This was despite that AMF communities across *low* plots appeared more divergent than across *high* plots (Fig. 2b) and that we observed differences in relation to the aggregation patterns (Fig. 1). In Grünfeld et al. (2019), we had observed pronounced differences in root colonization between *high* and *low* plots across forests in the same general area, but we had worked at a relatively larger spatial scale. AMF can grow vegetatively to distances of about 50 cm (Klironomos and Moutoglis 1999), but they could also potentially disperse by other means such as air and animal vectors (Egan et al. 2014). We may have thus missed the relevant spatial scale, or differences in relation to the mycorrhizal state of the canopy affect percentage colonization to a greater degree than they affect AMF community composition.

We compare and rank relative effect sizes of drivers of AMF community composition operating at a small spatial scale (as compared to soil properties and climatic variables that operate at larger scales) that have rarely been addressed simultaneously. Several authors such as Dumbrell et al. (2010b)have highlighted the need to better understand stochastic processes in AMF, and our study presents a ranking exercise which contributes towards satisfying that need.

## Supplementary information

Below is the link to the electronic supplementary material.

Supplementary file1 (DOCX 386 KB)

## Data Availability

The data will be made available upon publication.

## Code availability

We attach the code in the supplement.
