# Supplementary material for: Disentangling the relative importance of spatio-temporal parameters and host specificity in shaping arbuscular mycorrhizal fungus communities in a temperate forest
Source: Mycorrhiza. 2021 Jul 19;31(5):589–98. doi: 10.1007/s00572-021-01041-6 (PMC8484218; doi:10.1007/s00572-021-01041-6)
Supplement: Supplementary file 1 — Supplementary file1 (DOCX 386 KB) [file 572_2021_1041_MOESM1_ESM.docx]

**Supporting Information**

Article title: Disentangling the relative importance of spatio-temporal parameters and host specificity in shaping arbuscular mycorrhizal fungus communities in a temperate forest

Leonie Grünfeld, Magkdi Mola, Monika Wulf, Stefan Hempel, Stavros D Veresoglou*

*Freie Universität Berlin, Institut für Biologie, Altensteinstr. 6, D-14195 Berlin, Germany, Email: sveresoglou@zedat.fu-berlin.de, phone number: +49 30 838 53172

**Fig. S1** Schematic plan of the study site

**Fig. S2** Choosing an AM-coverage threshold

**Fig. S3** Richness statistics from all plots and harvests

**Fig. S4** PCA of AMF community data (first and second axes)

**Fig. S5** PCA of AMF community data after excluding outliers

**Table S1** AM plant cover and sampling information

**Table S2** Soil properties of the sampling site

**Table S3** OTU ID information

**Appendix I** Defining stochasticity

**Appendix II** Limitations of existing multivariate techniques in addressing spatio-temporal designs

**Appendix III** Statistical analyses


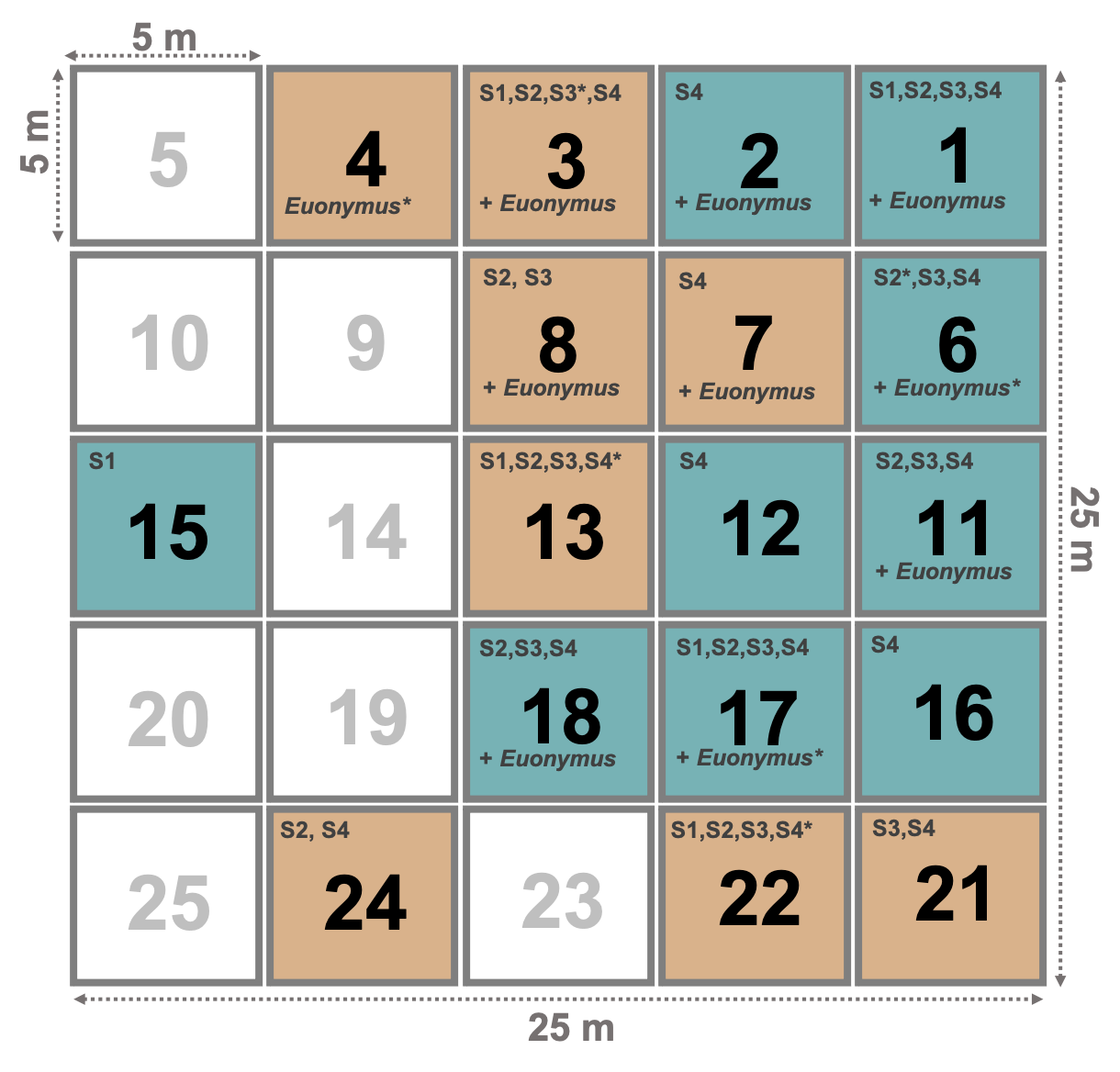


**Fig. S1** Schematic plan of the 25 5 m x 5 m plots labelled with study plot identity numbers (1-25) at the forest site. Green squares indicate plots with *high* (≥ 15 % AM plant cover) and brown squares indicate plots with *low* (< 15 % AM plant cover). White squares indicate plots where no root samples were collected. Root samples of *Hedera* are labelled S1 (May 2017), S2 (September 2017), S3 (May 2018), and S4 (September 2018) on respective plots; root samples of *Euonymus* collected in September 2018 only are noted. In general, two root samples from each of two individuals were collected with a minimum distance of 50 cm between them. Exceptions where only one individual was sampled, because no other plant was present, are indicated with an asterisk.


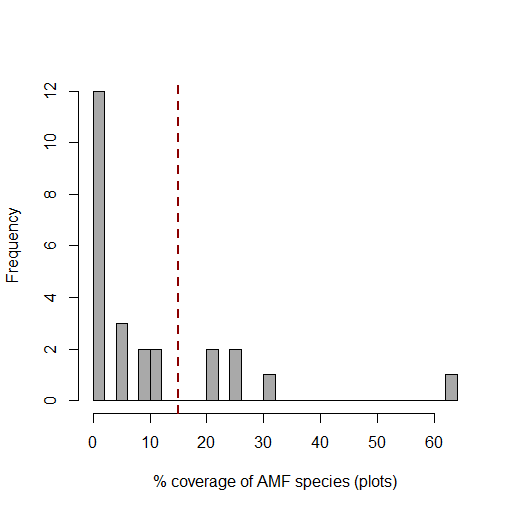


**Fig. S2** Histogram depicting the classification threshold of 15% in relation to AM-host coverage across plots. Plots with a relative coverage of AMF-associating woody species of over 15% were classified as *high* plots whereas those with a relative coverage of AMF-associating woody species of below 15% as *low*. The plot with a relative coverage of AMF-associating woody species of 64% was no outlier: That specific plot was only assayed for *Hedera* at the fourth harvest and the two samples had AMF richness of 11 and 9 respectively (richness estimates ranged between 4 and 17 OTUs per sample (median: 10 OTUs with the quartiles being 8 and 12). Additionally, in the ordination plots there was nothing special about the clustering patterns of these two samples.


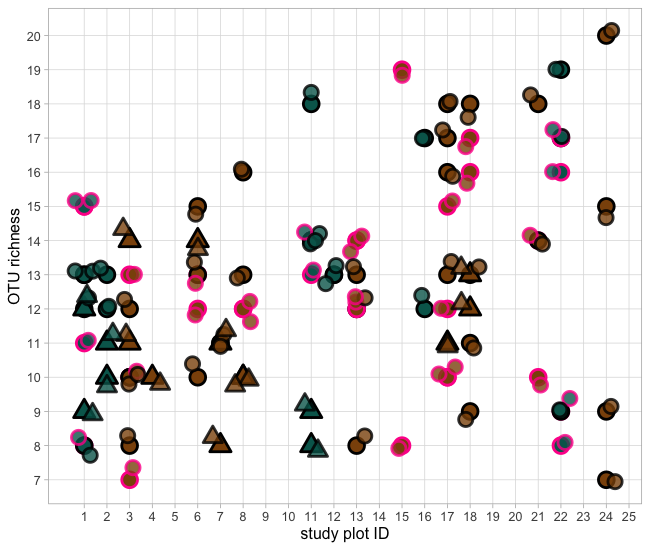


**Fig. S3** Richness based on numbers of OTUs from each harvest across sampling plots. Green symbols represent samples from *high* plots whereas brown represent *low* plots. A pink border was used for spring and a black border for autumn. Triangles represent samples of *Euonymus* whereas circles those of *Hedera*.


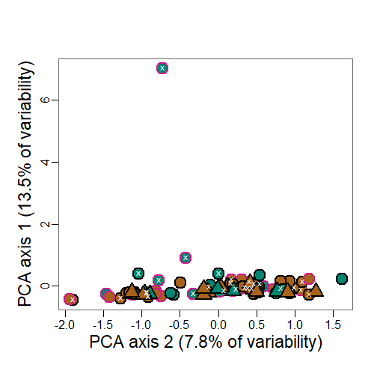


**Fig. S4** Principal component analysis of Hellinger transformed AMF community data with axis one explaining 13.5 % of variability. Green points represent samples from high plots whereas brown represent low plots. A pink border was used for spring and a black border for autumn. We used white “x” symbols to highlight samples taken over the first year. Triangles represent samples of *Euonymus* whereas circles those of *Hedera*. In main text Fig. 2a we display axes two and three because after excluding the outlier on axis one the rescaled variances for the three axes are 1.03% - axis 1, 7.84% - axis 2 and 6.88% - axis 3.


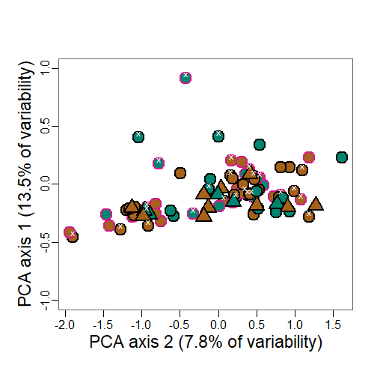


**Fig. S5** Principal component analysis of Hellinger transformed AMF community data with axis one explaining 13.5 % of variability (see Fig. S4) after the exclusion of outliers. Green points represent samples from high plots whereas brown represent low plots. A pink border was used for spring and a black border for autumn. We used white “x” symbols to highlight samples taken over the first year. Triangles represent samples of *Euonymus* whereas circles those of *Hedera*. In main text Fig. 2a we display axes two and three because after excluding the outlier in axis one the rescaled variances for the three axes are 1.03% - axis 1, 7.84% - axis 2 and 6.88% - axis 3. The take home message of this figure is that with the outlier included, the scores on axis 1 are driven by that outlier resulting in a strong correlation on that axis.

**Table S1** Visually assessed AMF-associating plant cover across the 25 plots presented in Fig. S1. We used a % cover threshold of 15% to classify them into *high* and *low* plots (see Fig. S2). Roots were collected from 17 of the 25 plots. The four harvests took place on 31 May 2017, 27 September 2017, 31 May 2018 and 25 September 2018. We progressively made the harvests from plots where the two species had a high relative abundance. This was done to avoid issues arising from the two plants becoming locally extinct.

| plot within site | % cover  of AM-associating woody plants | classification  AM plant cover | harvests where  *Hedera helix* was sampled | Sampling  *Euonymus europaea*  In 4th harvest |
| --- | --- | --- | --- | --- |
| 1 | 26 | High | 1-4 | Yes |
| 2 | 31 | High | 4 | Yes |
| 3 | 10 | Low | 1-4 | Yes |
| 4 | 1 | Low | NA | Yes |
| 6 | 6 | Low | 2-4 | Yes |
| 7 | 11 | Low | 4 | Yes |
| 8 | 5 | Low | 2-3 | Yes |
| 11 | 22 | High | 2-4 | Yes |
| 12 | 21 | High | 4 | No |
| 13 | 12 | Low | 1-4 | No |
| 15 | 1 | Low | 1 | No |
| 16 | 64 | High | 4 | No |
| 17 | 1 | Low | 1-4 | Yes |
| 18 | 2 | Low | 2-4 | Yes |
| 21 | 1 | Low | 3-4 | No |
| 22 | 26 | High | 1-4 | No |
| 24 | 2 | Low | 2,4 | No |

**Table S2** Soil pH (measured in 0.01m CaCl_2_) and nutrient levels of phosphorus, potassium, calcium and magnesium at the sampling site. Measurements were taken at three different soil depths by Wulf [1]. The soil constitutes a humid to waterlogged pseudogley [2]. Because we worked at a relatively small spatial scale we assumed soil characteristics to be consistent across plots.

| soil depth | pH | Exchangeable P [mg/100g] | K [mg/100g] | Ca [mg/100g] | Mg [mg/100g] |
| --- | --- | --- | --- | --- | --- |
| 0 - 5 cm | 4.6 | 2.8 | 11.2 | 139.6 | 9.8 |
| 5 - 10 cm | 5.1 | 1.3 | 5.9 | 168.2 | 8.5 |
| 10 - 20 cm | 5.6 | 0.9 | 3.6 | 188.5 | 7.8 |

**Table S3** OTU ID information. We accepted OTUs as belonging to Glomeromycota when they had > 97.5 % similarity and > 99 % coverage to entries in MaarjAM.

| OTU ID | Accession | Description | Max score | Total score | Query coverage | E-value | Max identity |
| --- | --- | --- | --- | --- | --- | --- | --- |
| OTU5 | HF568031 | Glomeraceae Glomus Varela-Cervero15 BG9 | 154 | 218 | 90.10% | 5.20E-80 | 85.50% |
| OTU6 | HF568033 | Glomeraceae Glomus Varela-Cervero15 BG9 | 158 | 222 | 90.30% | 2.50E-82 | 84.70% |
| OTU11 | HF568087 | Glomeraceae Glomus Varela-Cervero15 BG21 | 135 | 200 | 89.00% | 5.10E-69 | 82.30% |
| OTU14 | LT723796 | Glomeraceae Glomus sp. VTX00342 | 34 | 34 | 15.70% | 1.40E-10 | 82.90% |
| OTU19 | JN009464 | Diversisporaceae Diversispora sp. VTX00380 | 516 | 516 | 100.00% | 0 | 99.40% |
| OTU22 | HF568033 | Glomeraceae Glomus Varela-Cervero15 BG9 | 158 | 222 | 90.30% | 2.50E-82 | 84.70% |
| OTU24 | KC665641 | Glomeraceae Glomus Lopez-Garcia14 Glo166 | 76 | 76 | 33.30% | 6.80E-35 | 83.80% |
| OTU26 | HF568087 | Glomeraceae Glomus Varela-Cervero15 BG21 | 173 | 238 | 90.10% | 5.20E-91 | 85.90% |
| OTU27 | LT723796 | Glomeraceae Glomus sp. VTX00342 | 34 | 34 | 15.70% | 1.40E-10 | 82.90% |
| OTU28 | KJ959950 | Glomeraceae Glomus sp. VTX00219 | 479 | 479 | 100.00% | 0 | 97.70% |
| OTU32 | HF568038 | Glomeraceae Glomus Varela-Cervero15 BG9 | 169 | 228 | 87.60% | 1.10E-88 | 85.40% |
| OTU34 | KC708365 | Claroideoglomeraceae Claroideoglomus sp. VTX00193 | 427 | 427 | 100.00% | 0 | 94.70% |
| OTU36 | FN869852 | Archaeosporaceae Archaeospora Aca VTX00338 | 473 | 473 | 100.00% | 0 | 98.80% |
| OTU37 | HF568087 | Glomeraceae Glomus Varela-Cervero15 BG21 | 149 | 216 | 91.50% | 4.10E-77 | 83.70% |
| OTU39 | LT934587 | Glomeraceae Glomus sp. | 158 | 225 | 93.90% | 2.50E-82 | 83.30% |
| OTU41 | HF568087 | Glomeraceae Glomus Varela-Cervero15 BG21 | 176 | 238 | 89.40% | 9.60E-93 | 85.80% |
| OTU42 | HF568058 | Glomeraceae Glomus Varela-Cervero15 BG13 | 159 | 221 | 92.60% | 6.60E-83 | 84.00% |
| OTU45 | HF568021 | Glomeraceae Glomus Varela-Cervero15 BG7 | 122 | 190 | 87.00% | 1.80E-61 | 81.50% |
| OTU47 | KC665641 | Glomeraceae Glomus Lopez-Garcia14 Glo166 | 82 | 82 | 33.30% | 2.40E-38 | 85.10% |
| OTU50 | HF568040 | Glomeraceae Glomus Varela-Cervero15 BG9 | 174 | 238 | 86.50% | 1.40E-91 | 87.70% |
| OTU52 | HF568040 | Glomeraceae Glomus Varela-Cervero15 BG9 | 183 | 247 | 86.50% | 8.70E-97 | 88.70% |
| OTU54 | KC665641 | Glomeraceae Glomus Lopez-Garcia14 Glo166 | 85 | 85 | 33.30% | 4.50E-40 | 85.80% |
| OTU55 | HF568087 | Glomeraceae Glomus Varela-Cervero15 BG21 | 165 | 230 | 88.80% | 2.20E-86 | 85.30% |
| OTU58 | HF568034 | Glomeraceae Glomus Varela-Cervero15 BG9 | 164 | 220 | 87.90% | 8.50E-86 | 84.80% |
| OTU59 | HF568038 | Glomeraceae Glomus Varela-Cervero15 BG9 | 152 | 152 | 73.90% | 7.40E-79 | 83.90% |
| OTU60 | FN429106 | Glomeraceae Glomus VeGlo13 VTX00153 | 495 | 495 | 100.00% | 0 | 98.30% |
| OTU62 | AB746997 | Claroideoglomeraceae Claroideoglomus Yoshimura13b Glo17 | 435 | 435 | 100.00% | 0 | 94.30% |
| OTU63 | JQ246044 | Glomeraceae Glomus sp. VTX00359 | 79 | 79 | 67.60% | 1.30E-36 | 76.70% |
| OTU64 | LT934587 | Glomeraceae Glomus sp. | 152 | 214 | 92.10% | 7.50E-79 | 82.70% |
| OTU65 | LN900648 | Glomeraceae Glomus sp. VTX00096 | 477 | 477 | 100.00% | 0 | 97.30% |
| OTU66 | HF568040 | Glomeraceae Glomus Varela-Cervero15 BG9 | 166 | 227 | 87.00% | 6.00E-87 | 87.50% |
| OTU67 | AJ854097 | Archaeosporaceae Archaeospora sp. VTX00006 | 450 | 487 | 100.00% | 0 | 99.30% |
| OTU70 | EU417640 | Glomeraceae Glomus Sciaphila ledermannii symbiont VTX00166 | 498 | 498 | 100.00% | 0 | 98.50% |
| OTU71 | LN623488 | Paraglomeraceae Paraglomus MO-P2 VTX00433 | 125 | 195 | 87.60% | 3.20E-63 | 81.40% |
| OTU72 | LT983627 | Glomeraceae Glomus SS-G1 VTX00448 | 248 | 313 | 91.20% | 2.00E-134 | 92.30% |
| OTU73 | LN827079 | Glomeraceae Glomus sp. VTX00344 | 466 | 466 | 100.00% | 0 | 96.90% |
| OTU74 | HF568033 | Glomeraceae Glomus Varela-Cervero15 BG9 | 160 | 221 | 90.10% | 1.70E-83 | 84.90% |
| OTU75 | HF569099 | Gigasporaceae Scutellospora Varela-Cervero15 BS2 | 140 | 205 | 91.50% | 6.50E-72 | 82.30% |
|  |  |  |  |  |  |  |  |

**Appendix I** Defining stochasticity

The term stochasticity often has been used in the literature synonymously with variability [3]. Shoemaker *et al.* [3] propose narrowing the definition of stochasticity to processes that can be represented in a probabilistic way defined by their parameters (e.g. mean, variance, and skew) and those authors distinguish three forms of stochasticity: demographic stochasticity, environmental stochasticity, and measurement error. Based on this definition it is possible that the aggregate of community variance that is explained by deterministic processes and stochasticity falls below 100%.

In our study we define stochasticity in relation to the proportion of variance that is not explained by deterministic processes (i.e., deterministic processes and variance together explain 100% of community variance). We do so because arbuscular mycorrhizal associations represent complex systems which we do not understand sufficiently to effectively model processes such as demographic stochasticity (priority effects and succession, for example, have been poorly defined for AMF systems) which could have led us to seriously underestimate stochasticity. We thus describe stochasticity here as the fraction of community variance that is not explained by deterministic processes [4].

To show a few examples of the proportion of stochasticity that authors often find in AMF communities we carried out a literature search. We used the key words "deterministic" AND "arbuscular" in the Web of Knowledge on 30 June 2020. The search yielded 9 results which we screened for studies that contained figures on variance explained following a variance partitioning exercise. There were four such studies, which we list below:

| study | variance explained by deterministic factors |
| --- | --- |
| Maciel Rabelo Pereira *et al.* 2020 Journal of Biogeography, [5] | 9% |
| Rasmussen *et al.* 2018 New Phytologist [6] (Caruso 2018 New Phytologist Commentary, [7]) | 7-25% |
| Kohout *et al.* 2015 Molecular Ecology [8] | 39.3% |
| Dumbrell *et al.* 2010 ISME Journal [9] | 68.5% |

**Appendix II** Limitations of existing multivariate techniques in addressing spatio-temporal designs and how we addressed them

Most studies addressing the relative role of deterministic and stochastic processes in structuring communities engage in variance partitioning with the aim to explain as much variance as possible (e.g. [10]). Accuracy of such estimates depends strongly on the degree to which the underlying ordination model captures the experimental design. For example, assaying a plot at multiple instances in time represents a violation of independence and potentially can be addressed through a repeated-measures design in which case plot will be the unit of analysis (i.e. *Subject*) whereas time is the within-subject factor (e.g. [11]). Determining between-subject and within-subject sources of variance is critical in the case of variance partitioning because through this step it becomes apparent that each group will be compared against a unique fraction of unexplained variance (e.g., within-subjects unexplained variance). Many of the studies addressing spatio-temporal designs, however, incorrectly assume that iterative harvests of a single plot are independent of each other.

Even though there are several techniques to address spatial autocorrelation in ordination analyses, to the best of our knowledge the only multivariate technique that works for temporal constraints is that of Palmer *et al.* [11], which was specifically proposed for split plot designs. To minimize the assumptions of our analyses we plot our data with a PCA (i.e., meaning that we do not propose any underlying model; main text Fig. 2a) and then calculate distributions of pairwise Bray-Curtis distances (Fig. 2b). To back up our analysis we fit a redundancy analysis model (Appendix III) in which we address temporal constraints by restricting permutations (and thus calculation of resulting *P* values) to only be within plots. This approach may be an improvement compared to assuming full independence.

Our study was subject to some limitations which we raise here. First, we did not assay soil properties and assumed that the abiotic parameters throughout the site were homogenous. To this end we included in our analysis spatial corrections which should have accounted for spatial autocorrelations in soil properties. Second, to simultaneously address all factors we sacrificed the number of levels we considered. We worked with two plant species over two years and two seasons. Some of the results may be idiosyncratic because of this choice. Third, we only assayed two AM plant species, which were those that showed sufficient abundance across the target forest site. One of them (*Euonymus)* could only be collected at the last harvest. This was done because there were only a few individuals of *Euonymus* in the forest site and their destructive harvest could modify meta-community dynamics of AMF species. As a result, to a certain degree the variance that we allocated to plant species was nested within the variance explained by season and year. To address this point, we only tested spatio-temporal parameters for *Hedera*, and in the cases where we included *Euonymus* in analyses we formulated the models we fitted so that there were several alternative ranks of predictors.

**Appendix III** Statistical analyses

Most of our statistics were descriptive. Ordination analyses were carried out after Hellinger transforming community data (for example, main text Fig. 2a) to avoid distortions of distances in the Euclidean space. To assay the distributions of Bray Curtis distances we randomly paired samples 9999 times (Fig. 2b). Relative abundance figures of AMF families were descriptive (Fig. 2c).

We carried out an Analysis of Variance on the RDA models that we fitted. The default settings of the command anova.cca in R, test hierarchically the terms of the RDA/CCA models and yield different results from when the predictors are fitted “simultaneously”. Additionally, the command does not offer possibilities to fully distinguish between within-factor and between factors variability (i.e., does not address repeated-measures designs). An alternative is to constrain permutations so that they occur within the subjects of the analysis (i.e., here the plots), which we applied. To address that our experimental design was not balanced and that predictors were fitted hierarchically we tested alternative formulations of the models after changing the rank of the predictors. We additionally included models with and without the predictor *plant species*. To address spatial constraints, we used the Principal Coordinates of Neighbourhood Matrix (PCNM) approach to summarize space into three spatial axes. The parameters *year*, spatial PCNM axes and *plant species* (in the cases when it was included) were significant regardless of their rank in the models. Season was never significant, whereas the predictor *AM coverage* was only occasionally significant. We attach specific results:

**Redundancy Analysis Statistics**

> anova(myrda, by="terms", permutations=9999, strata=as.factor(field$plot))

Permutation test for rda under reduced model

Terms added sequentially (first to last)

Blocks: strata

Permutation: free

Number of permutations: 9999

Model: rda(formula = data2 ~ AM_coverage + year + season + plant_species + PCNM1 + PCNM2 + PCNM3, data = field, scale = T)

Df Variance F Pr(>F)

AM_coverage 1 0.6071 1.5900 0.0002 ***

year 1 0.8247 2.1600 0.0009 ***

season 1 0.5360 1.4040 0.1974

plant_species 1 0.5651 1.4801 0.0203 *

PCNM1 1 0.5948 1.5580 0.1529

PCNM2 1 0.5199 1.3616 0.5799

PCNM3 1 0.5720 1.4982 0.0351 *

Residual 78 29.7804

---

Signif. codes: 0 ‘***’ 0.001 ‘**’ 0.01 ‘*’ 0.05 ‘.’ 0.1 ‘ ’ 1

> anova(myrda, by="terms", permutations=9999, strata=as.factor(field$plot))

Permutation test for rda under reduced model

Terms added sequentially (first to last)

Blocks: strata

Permutation: free

Number of permutations: 9999

Model: rda(formula = data2 ~ year + plant_species + AM_coverage + season + PCNM1 + PCNM2 + PCNM3, data = field, scale = T)

Df Variance F Pr(>F)

year 1 0.7991 2.0929 0.0011 **

plant_species 1 0.6027 1.5786 0.0132 *

AM_coverage 1 0.6111 1.6005 0.1137

season 1 0.5200 1.3619 0.2276

PCNM1 1 0.5948 1.5580 0.1459

PCNM2 1 0.5199 1.3616 0.5852

PCNM3 1 0.5720 1.4982 0.0344 *

Residual 78 29.7804

---

Signif. codes: 0 ‘***’ 0.001 ‘**’ 0.01 ‘*’ 0.05 ‘.’ 0.1 ‘ ’ 1

> myrda<-rda(data2~year+season+AM_coverage+PCNM1+PCNM2+PCNM3, data=field[1:71,], scale=T)

> anova(myrda, by="terms", permutations=9999, strata=as.factor(field$subplot)[1:71])

Permutation test for rda under reduced model

Terms added sequentially (first to last)

Blocks: strata

Permutation: free

Number of permutations: 9999

Model: rda(formula = data2 ~ year + season + AM_coverage + PCNM1 + PCNM2 + PCNM3, data = field[1:71, ], scale = T)

Df Variance F Pr(>F)

year 1 0.7230 1.5365 0.0364 *

season 1 0.5508 1.1706 0.3986

AM_coverage 1 0.7495 1.5929 0.0459 *

PCNM1 1 0.7039 1.4961 0.6544

PCNM2 1 0.4805 1.0212 0.6495

PCNM3 1 0.6796 1.4445 0.0937 .

Residual 64 30.1127

---

Signif. codes: 0 ‘***’ 0.001 ‘**’ 0.01 ‘*’ 0.05 ‘.’ 0.1 ‘ ’ 1

> anova(myrda, by="terms", permutations=9999, strata=as.factor(field$plot)[1:71])

Permutation test for rda under reduced model

Terms added sequentially (first to last)

Blocks: strata

Permutation: free

Number of permutations: 9999

Model: rda(formula = data2 ~ year + season + PCNM1 + PCNM2 + PCNM3 + AM_coverage, data = field[1:71, ], scale = T)

Df Variance F Pr(>F)

year 1 0.7230 1.5365 0.0338 *

season 1 0.5508 1.1706 0.4004

PCNM1 1 0.7231 1.5369 0.7505

PCNM2 1 0.4844 1.0294 0.1324

PCNM3 1 0.9037 1.9206 0.0587 .

AM_coverage 1 0.5023 1.0676 0.9381

Residual 64 30.1127

---

Signif. codes: 0 ‘***’ 0.001 ‘**’ 0.01 ‘*’ 0.05 ‘.’ 0.1 ‘ ’ 1

> anova(myrda, by="terms", permutations=9999, strata=as.factor(field$subplot)[1:71])

Permutation test for rda under reduced model

Terms added sequentially (first to last)

Blocks: strata

Permutation: free

Number of permutations: 9999

Model: rda(formula = data2 ~ PCNM1 + PCNM2 + PCNM3 + AM_coverage + season + year, data = field[1:71, ], scale = T)

Df Variance F Pr(>F)

PCNM1 1 0.7513 1.5968 0.0788 .

PCNM2 1 0.4173 0.8869 0.0788 .

PCNM3 1 0.8174 1.7372 0.0788 .

AM_coverage 1 0.5426 1.1533 0.0788 .

season 1 0.5270 1.1201 0.4780

year 1 0.8316 1.7674 0.0321 *

Residual 64 30.1127

---

Signif. codes: 0 ‘***’ 0.001 ‘**’ 0.01 ‘*’ 0.05 ‘.’ 0.1 ‘ ’ 1

**Repeated-measures ANOVA**

> summary(aov(S~AM_coverage+plant_species+season+year+ Error(as.factor(subplot)/as.factor(sampling)), data=field))

Error: as.factor(subplot)

Df Sum Sq Mean Sq F value Pr(>F)

AM_coverage 1 4.88 4.881 1.323 0.2725

plant_species 1 12.50 12.501 3.388 0.0905 .

season 1 2.57 2.575 0.698 0.4198

year 1 20.35 20.346 5.515 0.0368 *

Residuals 12 44.27 3.689

---

Signif. codes: 0 ‘***’ 0.001 ‘**’ 0.01 ‘*’ 0.05 ‘.’ 0.1 ‘ ’ 1

Error: as.factor(subplot):as.factor(sampling)

Df Sum Sq Mean Sq F value Pr(>F)

plant_species 1 51.06 51.06 7.281 0.0138 *

season 1 7.73 7.73 1.103 0.3062

year 1 4.21 4.21 0.601 0.4474

Residuals 20 140.25 7.01

---

Signif. codes: 0 ‘***’ 0.001 ‘**’ 0.01 ‘*’ 0.05 ‘.’ 0.1 ‘ ’ 1

Error: Within

Df Sum Sq Mean Sq F value Pr(>F)

plant_species 1 27.34 27.337 4.483 0.0398 *

Residuals 45 274.41 6.098

---

Signif. codes: 0 ‘***’ 0.001 ‘**’ 0.01 ‘*’ 0.05 ‘.’ 0.1 ‘ ’ 1

Warning message:

In aov(S ~ AM_coverage + plant_species + season + year + Error(as.factor(subplot)/as.factor(sampling)), :

Error() model is singular

**Indicator Species Analysis**

> ind.sp = multipatt(data2, as.factor(paste0(as.factor(field$plant_species), as.factor(field$AM_coverage))), control = how(nperm=9999), max.order=2, restcomb=c(1:6, 9:10))

Association function: IndVal.g

Significance level (alpha): 0.05

Total number of species: 34

Selected number of species: 5

Number of species associated to 1 group: 2

Number of species associated to 2 groups: 3

Number of species associated to 3 groups: 0

List of species associated to each combination:

Group Euonymus europaea #sps. 1

stat p.value

OTU70 0.826 2e-04 ***

Group Hedera helix #sps. 1

stat p.value

OTU19 0.644 0.0383 *

Group Euonymus europaea+Euonymus europaea #sps. 1

stat p.value

OTU2 0.854 0.0349 *

Group Hedera helix+Hedera helix #sps. 2

stat p.value

OTU8 0.904 0.0007 ***

OTU13 0.880 0.0027 **

---

Signif. codes: 0 ‘***’ 0.001 ‘**’ 0.01 ‘*’ 0.05 ‘.’ 0.1 ‘ ’ 1

**Supplementary References**

1. Wulf M (1992) Vegetationskundliche und ökologische Untersuchungen zum Vorkommen gefährdeter Pflanzenarten in Feuchtwäldern Nordwestdeutschlands. Schweizerbart Science Publishers, Stuttgart, Germany

2. Roeschmann G (1971) Die Böden der nordwestdeutschen Geest-Landschaft. Mitteilungen der Dtsch Bodenkundlichen Gesellschaft 13:151–231

3. Shoemaker LG, Sullivan LL, Donohue I, et al (2020) Integrating the underlying structure of stochasticity into community ecology. Ecology 101:1–17

4. De Vrieze J, De Mulder T, Matassa S, et al (2020) Stochasticity in microbiology: managing unpredictability to reach the Sustainable Development Goals. Microb Biotechnol 13:829–843

5. Maciel Rabelo Pereira C, López-García Á, da Silva DKA, et al (2020) Tropical forest type influences community assembly processes in arbuscular mycorrhizal fungi. J Biogeogr 47:434–444

6. Rasmussen PU, Hugerth LW, Blanchet FG, et al (2018) Multiscale patterns and drivers of arbuscular mycorrhizal fungal communities in the roots and root-associated soil of a wild perennial herb. New Phytol 220:1248–1261

7. Caruso T (2018) Disentangling the factors shaping arbuscular mycorrhizal fungal communities across multiple spatial scales. New Phytol 220:954–956

8. Kohout P, Doubková P, Bahram M, et al (2015) Niche partitioning in arbuscular mycorrhizal communities in temperate grasslands: A lesson from adjacent serpentine and nonserpentine habitats. Mol Ecol 24:1831–1843

9. Dumbrell AJ, Nelson M, Helgason T, et al (2010) Relative roles of niche and neutral processes in structuring a soil microbial community. ISME J 4:337–345

10. Horn S, Caruso T, Verbruggen E, et al (2014) Arbuscular mycorrhizal fungal communities are phylogenetically clustered at small scales. ISME J 8:2231–2242

11. Palmer MW, McGlinn DJ, Westerberg L, Milberg P (2008) Indices for detecting differences in species composition: Some simplifications of RDA and CCA. Ecology 89:1769–1771
